# Severe mitral regurgitation due to mitral leaflet aneurysm diagnosed by three-dimensional transesophageal echocardiography: a case report

**DOI:** 10.1186/s12872-016-0413-1

**Published:** 2016-11-22

**Authors:** Takao Konishi, Naohiro Funayama, Tadashi Yamamoto, Daisuke Hotta, Kenjiro Kikuchi, Katsumi Ohori, Hiroshi Nishihara, Shinya Tanaka

**Affiliations:** 1 Department of Cardiology, Hokkaido Cardiovascular Hospital, 1-30, West 13, South 27, Chuou-ku, Sapporo, 064-8622 Japan; 2 Department of Cardiovascular Surgery, Hokkaido Cardiovascular Hospital, Sapporo, Japan; 3 Department of Cancer Pathology, Hokkaido University, Graduate School of Medicine, Sapporo, Japan

**Keywords:** Mitral leaflet aneurysm, Mitral regurgitation, Three-dimensional transesophageal echocardiography, Case report

## Abstract

**Background:**

A small mitral valve aneurysm (MVA) presenting as severe mitral regurgitation (MR) is uncommon.

**Case presentation:**

A 47-year-old man with a history of hypertension complained of exertional chest discomfort. A transthoracic echocardiogram (TTE) revealed the presence of MR and prolapse of the posterior leaflet. A 6-mm in diameter MVA, not clearly visualized by TTE, was detected on the posterior leaflet on a three-dimensional (3D) transesophageal echocardiography (TEE). The patient underwent uncomplicated triangular resection of P2 and mitral valve annuloplasty, and was discharged from postoperative rehabilitation, 2 weeks after the operation. Histopathology of the excised leaflet showed myxomatous changes without infective vegetation or signs of rheumatic heart disease.

**Conclusions:**

A small, isolated MVA is a cause of severe MR, which might be overlooked and, therefore, managed belatedly. 3D TEE was helpful in imaging its morphologic details.

**Electronic supplementary material:**

The online version of this article (doi:10.1186/s12872-016-0413-1) contains supplementary material, which is available to authorized users.

## Background

Mitral valve aneurysm (MVA), a rare disorder first described by Saphir and Leroy in 1948 [1], is usually a complication of infective endocarditis [2–7]. Other causes of this disorder are rheumatic heart disease, Marfan’s syndrome [8], aortic regurgitation and hypertrophic cardiomyopathy. Furthermore, the reports of surgical case have usually described MVA 15 to 30 mm in diameter [5, 7, 9–14]. The complications of MVA includes expansion, perforation and severe valvular regurgitation. An early diagnosis followed by surgical treatment is of critical importance to reduce the rate of these complications. We report a 6-mm in diameter, isolated aneurysm of a mitral leaflet in a man who presented with severe mitral regurgitation (MR).

## Case presentation

**Fig. 1:**
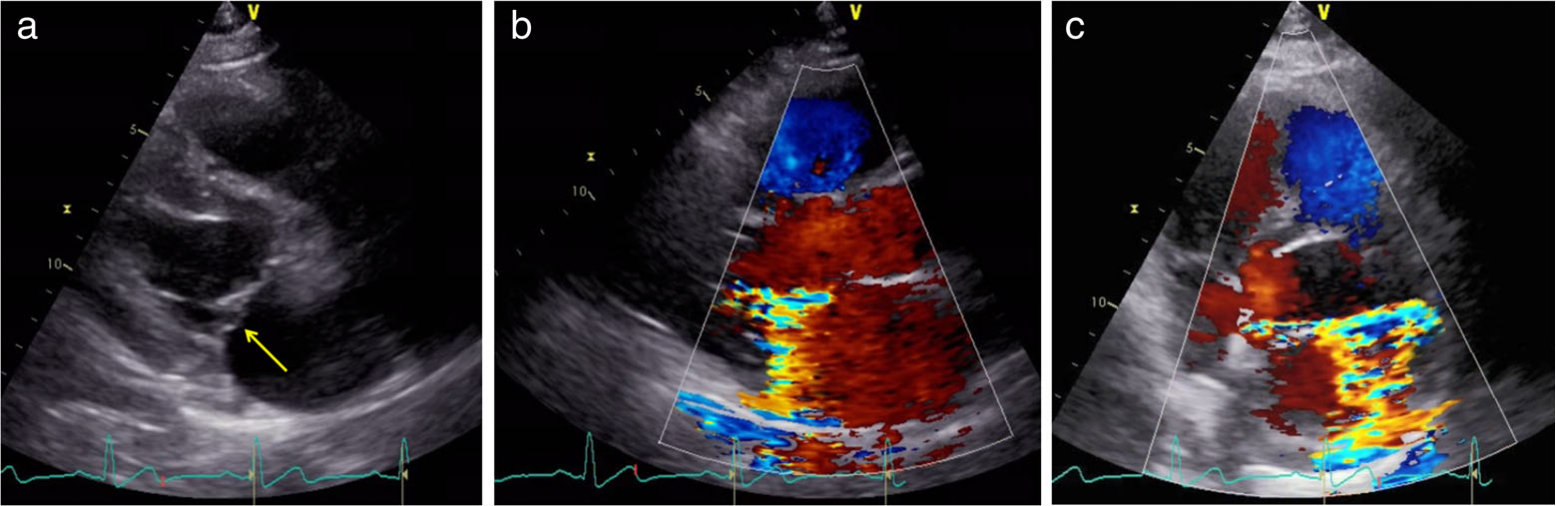
Transthoracic echocardiogram. **a**, **b** and **c**. Parasternal long **a** and **b** and short **c** axis view of the posterior mitral valve leaflet slightly protruding into the left atrium (*arrow*) with severe eccentric mitral regurgitation

**Fig. 2:**
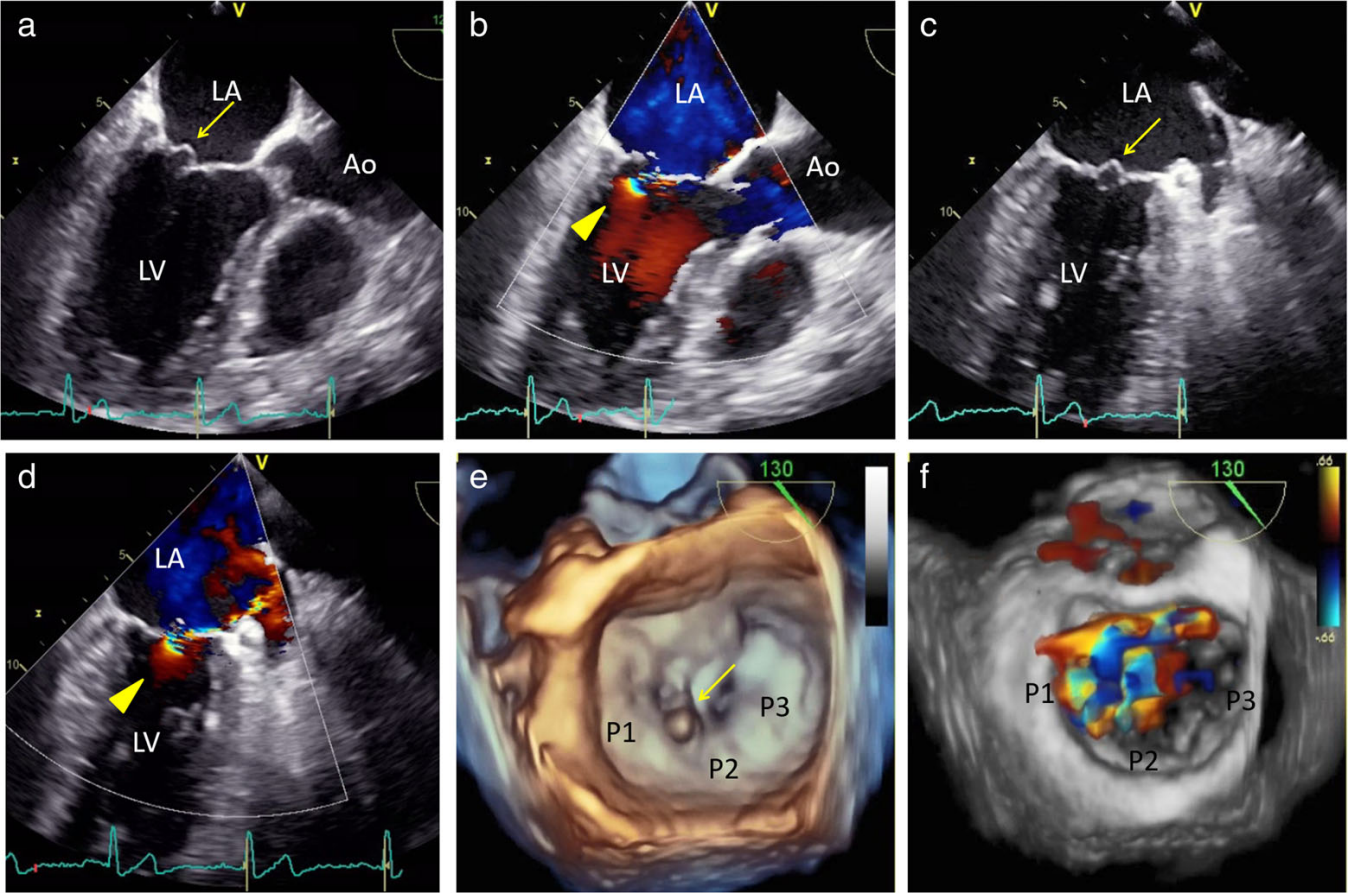
Transoesophageal echocardiogram. **a** and **c**. Part of the posterior leaflet protrudes into the left atrium at a 129° (**a**) and 63° (**c**) angle in early systole (arrows). LA = left atrium; LV = left ventricle; Ao = Aorta. **b** and **d**. The colour Doppler images shows a considerably large proximal isovelocity surface area (PISA) (arrowheads) and a prominently eccentric mitral regurgitation at a 129° (**b**) and 63° (**d**) angle in end-systole. **e**. Three-dimensional transoesophageal echocardiogram, observed from the left atrium towards the mitral valve annulus, showing the 6-mm in diameter aneurysm on the posterior leaflet (arrow). The aneurysm was located at mid-posterior leaflet (P2) near lateral-posterior leaflet (P1). **f**. Three-dimensional full-volume colour acquisition of the mitral valve illustrating the spatial relationship between the MVA and the MR jet

**Fig. 3:**
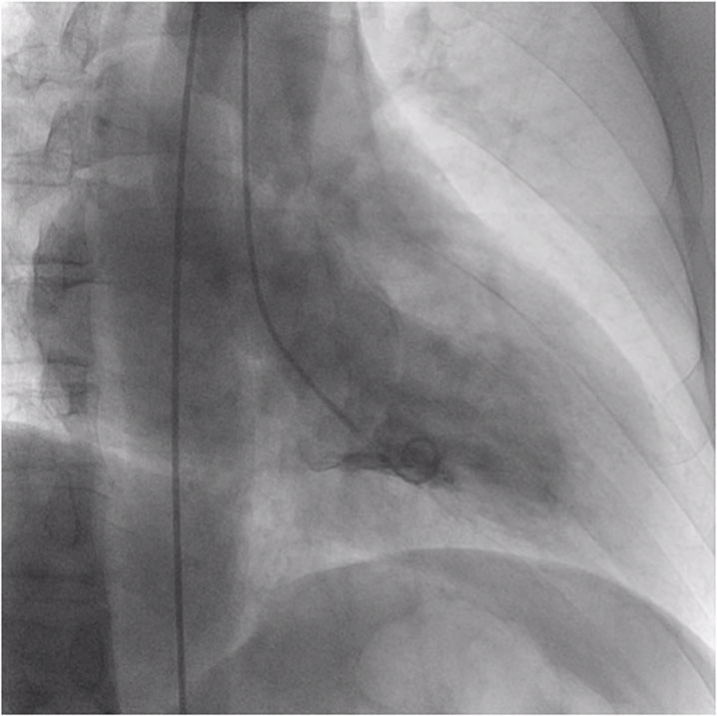
Left ventriculography. Right anterior oblique, 30° view of grade 3 to 4 mitral regurgitation (Seller’s grading)


A 47-year-old man with a 10-year history of hypertension complained of exertional chest discomfort. On physical examination, he was alert, his height and body weight were 168.5 cm and 78 kg, respectively, body temperature 36.3 °C, systemic blood pressure 152/82 mmHg and heart rate 56 bpm. The percutaneous oxygen saturation on room air was 97%. A III/VI systolic murmur was heard at the apex, radiating to the left axilla, without manifestation of cardiac decompensation. He had no sign of Marfan’s syndrome. The laboratory tests revealed a 4.0 × 10^3^/mm^3^ white blood cell count with 66% granulocytes and 27% lymphocytes, a 20.4 × 10^4^/mm^3^ platelet count, 15.7 g/dl haemoglobin concentration, and 0.03 mg/dl C-reactive protein serum concentration. A chest roentgenogram showed a 48% cardiothoracic ratio, and the 12-lead electrocardiogram showed a high QRS voltage in the lateral precordial leads. A transthoracic echocardiogram (TTE) revealed MR with prolapse of the posterior leaflet (Fig. 1a-c) without vegetation or aortic regurgitation. The echocardiographic left atrial and left ventricular (LV) dimensions and LV ejection fraction are shown in Table 1A. A two-dimensional transoesophageal echocardiogram (TEE) showed mitral valve prolapse (Fig. 2a and c) and MR with a significant proximal isovelocity surface area (PISA) (Fig. 2b and d). On three-dimensional (3D) TEE, a 6-mm in diameter aneurysm was present on the posterior mitral valve leaflet, which was not clearly visible on TTE (Fig. 2e and Additional file: Video S1). 3D full-volume colour TEE was also useful for illustrating the spatial relationship between the MVA and the MR jet (Fig. 2f and Additional file: Video S2). A LV angiogram (Fig. 3 and Additional file: Video S3) showed grade 3–4 MR and coronary angiograms showed no significant stenosis. The pressures measured during right heart catheterization are shown in Table 1B. Cardiac index was within normal limits (Table 1B).

**Table 1:** Transthoracic echocardiographic and right heart catheterisation measurements

A. Transthoracic echocardiogram	
Left ventricular ejection fraction, %	61
Diameters, mm	
Left atrial	47
Left ventricular (end-diastolic)	55
B. Right heart catheterization	
Pressures, mmHg	
Right	
Atrial	10 (1–5)
Ventricular	
End-systolic	36 (15–30)
End-diastolic	4 (1–7)
Pulmonary	
Arterial	
Systolic	35 (15–30)
Diastolic	18 (4–12)
Mean	24 (9–18)
Capillary wedge	18 (4–12)
Cardiac index, l/min/m^2^	3.2 (2.5–4.0)

Normal values are shown in parentheses

Additional file 1: **Video S1**. 3D TEE showing a 6-mm in diameter aneurysm on the posterior mitral valve leaflet.



Additional file 2: **Video S2**. 3D full-volume colour TEE illustrating the spatial relationship between the MVA and the MR jet.



Additional file 3: **Video S3**. LV angiogram showing grade 3–4 MR.


**Fig. 4:**
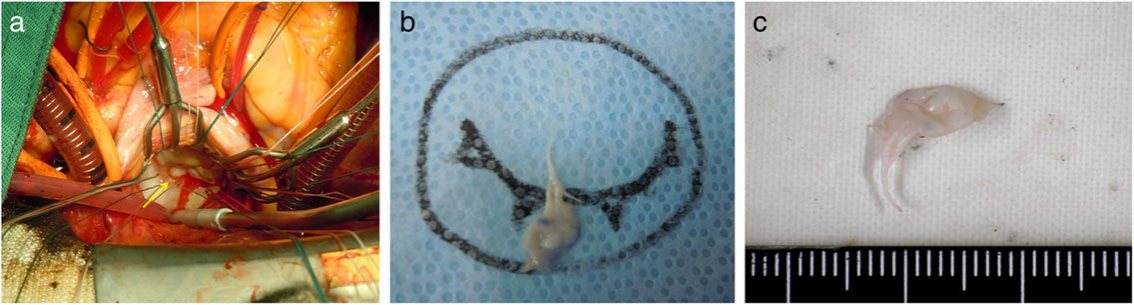
Intraoperative aspect and histopathology of the posterior mitral valve leaflet. **a**. Intraoperative aspect of the MVA on the posterior mitral leaflet (*arrow*). **b**. The excised MVA was a part of the mid-posterior leaflet (P2). **c**. The excised MVA measured 6 mm in diameter

**Fig. 5:**
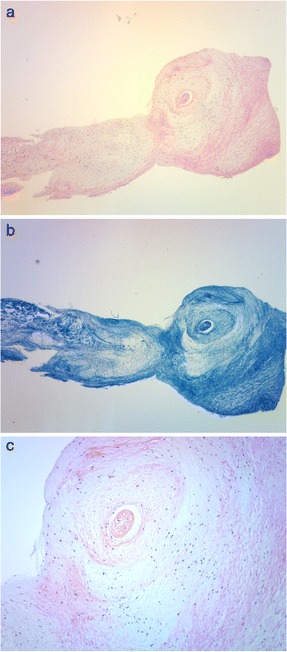
Histopathology of the mitral valve aneurysm. **a** and **b.** Low-power (X 5 original magnification) microphotograph of the mitral valve aneurysm showing prominent myxomatous changes observed after **a** haematoxylin and eosin, and **b** Masson Trichrome staining. **c**. Low-power (X 10 original magnification) microphotograph showing no infiltration by inflammatory cells or active endocarditis in the posterior mitral leaflet

The patient underwent open-heart, triangular resection of the P2 segment of the mitral valve and annuloplasty with a 28-mm Séguin ring. Intraoperative inspection confirmed the presence of an aneurysm on the posterior mitral leaflet (Fig. 4a-c). High- and low-power histopathologic microphotographs showed myxomatous degeneration, without active mitral valve endocarditis or inflammatory cellular infiltration of the posterior leaflet (Fig. 5a-c). The postoperative course was uneventful and the chest discomfort resolved during the postoperative rehabilitation program. The patient was discharged from postoperative rehabilitation, 2 weeks after the operation, and has remained free from significant MR over a 2-year follow-up.

## Discussion

This case illustrates the rare characteristics of an isolated, non-infective MVA, which, albeit small, caused severe MR. This observation, to the best of our knowledge, has not been previously reported in the English language medical literature.

A MVA is a discrete bulge of the mitral leaflet toward the left atrium expanding during systole and collapsing during diastole [15]. Its formation is associated with congenital, structural diseases of the connective tissues, including Marfan’s syndrome [8], mitral valve prolapse [15], LV outflow tract obstruction, hypertrophic cardiomyopathy [16, 17] and bicuspid or quadricuspid aortic valves [18, 19]. From these predisposing disorders, MVA is formed by acquired factors, such as rheumatic fever, Libman-Sachs endocarditis and, in most cases, infective endocarditis [2, 3, 20–22]. It is usually detected on the anterior leaflet, due to infective endocarditis caused by an aortic regurgitant jet striking the leaflet [9, 21, 22]. In this patient, however, it was on the posterior leaflet, in absence of regurgitant jet. In addition, the histopathology showed myxomatous degeneration without active endocarditis or inflammatory cells in the excised valve specimen (Fig. 5a-c). This supports our hypothesis that, in this patient, the prolonged hypertensive LV pressure overload, besides the congenital structural weakness secondary to myxomatous degeneration, caused the development of an isolated MVA of the posterior mitral leaflet in absence of infection.

The diagnosis of a small MVA, as was discovered in this patient, is often challenging, because its 3 dimensions are difficult to detect and identify on TTE. The superior performance of 3-dimensional TEE in clarifying the anatomical details of MVA has been reported [10, 23]. Since they may cause serious complications, such as systemic embolization, leaflet perforation, and recurrent infective endocarditis [21, 24, 25], their prompt diagnosis and surgical management is of utmost importance. In this patient, 3-dimensional TEE showed a small MVA, which could not be detected by TTE. This case also highlights the severity of MR caused by a MVA as small as 10 mm in diameter.

## Conclusions

Small, isolated MVA causing significant MR are rare and may be neither visible on TTE nor managed in a timely fashion. Regardless of their size, MVA can cause severe MR. 3D TEE is an effective means of detecting their presence.

## Abbreviations

3D: Three-dimensional
MR: Mitral regurgitation
MVA: Mitral valve aneurysm
TEE: Transesophageal echocardiography
TTE: Transthoracic echocardiography.
